# Inhibitory effect of deltorphin-II on development of malaria in *Plasmodium berghei*-infected mice

**DOI:** 10.5281/zenodo.10870022

**Published:** 2015-03-23

**Authors:** Garasiya A. Ajitbhai, Prati P. Singh, Mukesh Kumar, Rajinder Singh, Vandana Dhiman

**Affiliations:** 1 Department of Pharmacology and Toxicology, National Institute of Pharmaceutical Education and Research (NIPER), S.A.S. Nagar, Mohali-160 062, Punjab, India; 2 Department of Pharmacy, Manav Bharti University, Solan, HP, India

## Abstract

**Background:**

Drug resistance has been one of the main obstacles in the fight against vector-borne infectious diseases. Among these diseases, malaria represents a serious public health challenge, mainly in the tropics, where vector-favourable climates are a crucial factor. Each of the various anti-malarial drugs currently used against this disease, such as quinolones, sulphonamides and artemisinins are inadequate and new strategies are required. Peptides are known to have a huge number of biological effects. Antimicrobial peptides (AMPs) have been proven to be effective against bacterial, fungal and viral infections. This study explored the effect of the peptide ‘deltorphin-II’ in *Plasmodium berghei-*infected mice.

**Materials and Methods:**

Mean percentage parasitaemia was calculated by studying infected erythrocytes after microscopic examination of 10^4^ erythrocytes from infected mice on days 4, 7, 10, 14 and 21 after infection in all groups. **Results.** Deltorphin-II shows maximum activity at a dose of 0.8 mg/kg/day from day 4 to day 21. Pre-treatment of infected mice with naltriben abrogates the deltorphin-II-mediated effect.

**Conclusion:**

Deltorphin-II inhibits the development of malaria, most probably via activation of the δ_2_ receptor.

## 1 Introduction

Malaria is a vector-borne infectious disease caused by parasitic protozoans of the genus *Plasmodium*, transmitted by *Anopheles* mosquitoes. Five *Plasmodium* species cause malaria in humans: *P. falciparum*, *P. vivax*, *P. ovale*, *P. malariae* and *P. knowlesi* [1]. It is a disease that can be treated in just 48 hours, yet, it can cause fatal complications if the diagnosis and treatment are delayed. Malaria is the fifth-most common cause of death from infectious diseases worldwide (after respiratory infections, HIV/AIDS, diarrheal diseases and tuberculosis) and the second-most common in Africa after HIV/AIDS. The indirect costs of malaria include lost productivity or income associated with illness or death. Several strategies have been tested to combat this disease [2]. For decades, drug resistance has been one of the main obstacles in the fight against malaria. It has been documented in three of the five malaria species known to affect humans in nature: *P. falciparum*, *P. vivax* and *P. malariae* [3]. With the onset of drug-resistant *Plasmodium* parasites, new strategies are required to combat this widespread disease. Host defence has a major antiparasitic effect against any spontaneously generated drug-resistant mutant. Malarial parasites must contend not only with the anti-malarial drug concentrations but also with host immunity, which can considerably reduce the emergence and spread of resistance. Host immunity kills parasites regardless of their anti-malarial resistance and reduces the probability of parasite survival (independent of drugs) at all stages of the transmission cycle. Previous studies have also shown the immunomodulatory effects of opioids on induction of acquired immunity during microbial infections [4]. Peptides are known to have a huge number of biological effects, and antimicrobial peptides (AMPs) are proven to be effective against bacterial, fungal and viral infections. These antimicrobial peptides provide the initial line of defence against infections in higher eukaryotes [5]. A group of linear heptapeptides, deltorphins from skin extracts of frogs of the genus *Phyllomedusa*, have a higher selectivity and affinity for delta opioid receptors [6]. Deltorphin analogues have a number of pharmacological effects, including stimulating angiogenesis [7], post-translational amino acid racemisation [8], enhancing extracellular levels of dopamine [9] and simulating the cardio protective effect of ischemic preconditioning [10], but the effect of deltorphin-II on malaria infection has not been studied yet. Thus, based on the literature supporting antimicrobial properties of peptides and involvement of the delta receptor in immunity, deltorphin-II was explored for anti-malarial activity against *P. berghei*.

## 2 Materials and methods

### 2.1 Animals and parasites

Swiss male mice (*Mus musculus*) weighing 20±2 g, supplied by the Central Animal Facility, National Institute of Pharmaceutical Education and Research, Mohali, India, were used in all experiments. Animals were kept in temperature- (22-24°C) and light- (12 hr on/off) controlled rooms and provided with standard animal feed and water. All experiments were carried out in accordance with the Guidelines for Care and Use of Animals in Scientific Research, Indian National Science Academy, New Delhi, India, as adapted and decreed by the Institutional Animal Ethics Committee. The lethal rodent malaria parasite (*P. berghei* ANKA) used for infecting the mice was obtained from the Central Drug Research Institute, Lucknow, India. They were cultured *in vivo* by transferring into mice once and later on were injected intraperitoneally (i.p.) to deliver a counted inoculum of 10^6^ IE (infected erythrocytes) per mouse.

### 2.2 Drugs and reagents

Deltorphin-II and naltriben methanesulfonate hydrate were obtained from Sigma-Aldrich. Deltorphin-II was solubilised in normal saline and naltriben methanesulfonate hydrate was solubilised in 10% DMSO in normal saline solution. Wright’s stain was obtained from *Himedia* Laboratories (Mumbai, India). Isopropyl alcohol, disodium hydrogen phosphate and potassium dihydrogen phosphate were obtained from Merck (India).

### 2.3 Preparation of reagents

*Preparation of Wright’s stain solution*

1 g of Wright’s stain was dissolved in 500 ml of methanol and kept undisturbed for at least two months in the dark for maturation before it was used.

*Preparation of staining buffer*

72 ml of 0.07 M disodium hydrogen phosphate solution and 28 ml of 0.07 M potassium dihydrogen phosphate solution were mixed, and the volume was made up to 1000 ml with triple glass-distilled water, with the pH adjusted to 7.2.

*Preparation of sodium citrate saline solution*

6.4 g tri-sodium citrate and 1.75 g sodium chloride were dissolved in 200 ml of triple glass-distilled water. The solution was then autoclaved at 15 psi pressure at 121°C for 15 min, followed by storage at 4°C until use.

### 2.4 Cryopreservation of malaria parasites

For cryopreservation of the parasites, infected blood from animals with parasitaemia between 5-30% was collected in tubes containing citrate buffer and centrifuged at 2000 rpm for 7 min. Supernatant was removed and pellets were transferred to cryo-vials containing equal volume (1:1 w/v) of sterile solution of glycerol (28%) and mannitol or sorbitol (4.2%) in normal saline, and stored quickly in liquid nitrogen at –196°C [11]. The cryo-vials were taken out from the liquid nitrogen and brought to 37°C. They were diluted with more sodium citrate saline solution and injected into each mouse so as to deliver a counted inoculum of 10^6^ IE per mouse.

### 2.5 Enumeration of parasitaemia

A thin blood smear made from a small drop of cut tail blood of a mouse was air-dried. The dried slides were placed on horizontal bars with the blood smear on the upper side. The smear was then covered with Wright’s stain poured at a constant flow rate from one side of the slide edge. After about 4-5 min it was covered with staining buffer for 12 min, washed with more staining buffer, air-dried and monitored under a light microscope. An appropriate area of a stained thin-blood film (about 200 cells/ field) was selected by monitoring under light microscopy. Starting from one end in the selected area, the erythrocytes were examined for the presence of parasites in the field, and then the slide was moved to another field in a particular direction, so that fields are not counted more than once. A total of 50 fields were observed (50 × 200 = 10,000). The observation of 10^4^ erythrocytes per slide was usually found to be adequate. The parasitaemia was expressed as percentage IE after microscopic examination of 10^4^ erythrocytes.

### 2.6 Blood schizonticidal action

A four-day suppression test was used for investigating anti -malarial activity of the test compound [12]. Seven different groups were created, namely: (1) untreated control, (2) chloroquine (CQ) (8 mg/kg/day)-treated, (groups 3-6) deltorphin-II (0.1, 0.2, 0.4 and 0.8 mg/kg/day, respectively)-treated and (7) naltriben (1 mg/kg)+deltorphin-II (0.8 mg/kg/day)-treated. The four concentrations of deltorphin-II were selected to confirm the nature of the effect, whether it is dose-dependent or not. In each group, six mice were used. On day 0, 2 hr after infection with *P. berghei*, drug treatments were given to the respective groups. All drug treatments were given orally (p.o*.*) using an oral feeding needle. The same drug treatments were repeated for three more days (days 1-3). In the seventh group, naltriben (1 mg/kg subcutaneously, s.c.) was given 30 min prior to the treatment of the highest dose (0.8 mg/kg/day) of deltorphin-II in infected mice on each of the four days post infection. From day 4 onwards (96 hr post infection), thin blood smears were prepared, stained with Wright’s stain and the percentage of parasitaemia was measured [13].

### 2.7 Data analysis

Values are expressed as mean±s.e.m. Percentage reduction in parasitaemia was calculated as described by Muregi *et al.* [14]. Comparison of different treatments was performed by one-way analysis of variance (ANOVA) followed by post hoc analysis by Tukey’s test, using Sigma Stat v. 3.5. *P*<0.05 was considered significant.

## 3 Results

**Figure 1.**
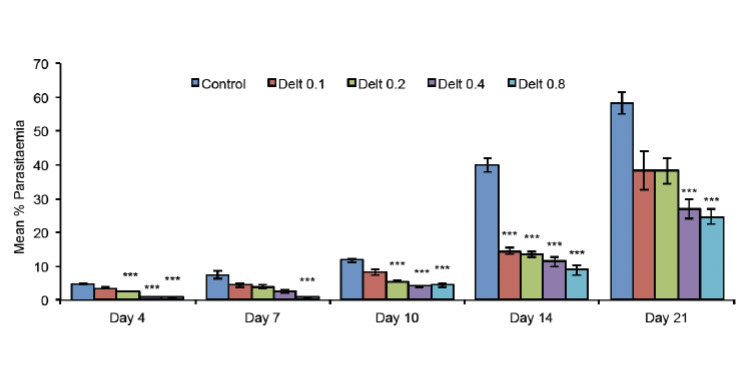
Effect of deltorphin-II (0.1, 0.2, 0.4 and 0.8 mg/kg/day×4, p.o.) on parasitaemia (%) in *P. berghei*-infected mice. Delt, deltorphin-II; p.o., per oral. All values are expressed as mean±s.e.m.^***^ :*P* <0.001 compared with untreated control.

**Figure 2.**
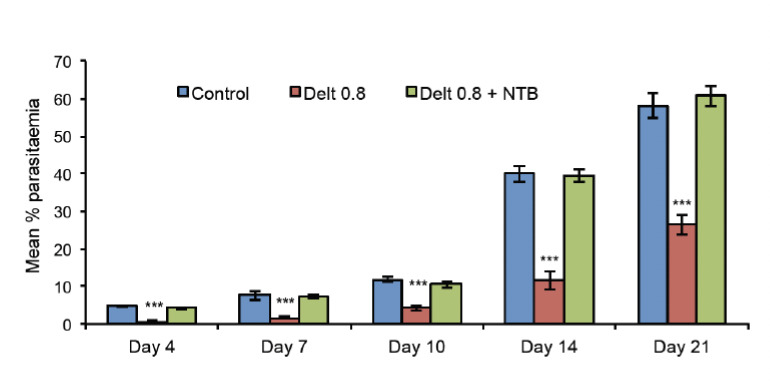
Effect of naltriben pre-treatment (1 mg/kg, s.c.) on parasitaemia (%) in deltorphin-II (0.8 mg/kg/day×4, p.o.)-treated *P. berghei*-infected mice. NTB, naltriben; Delt, deltorphin-II; p.o., per oral; s.c., subcutaneous. All values are expressed as mean±s.e.m.^***^ *P*<0.001 compared with untreated control.

The untreated control group showed a progressive increase in parasitaemia to 54.6% in 21 days. Treatment with CQ (8 mg/kg/day) was found to completely abolish the infection, with 100% reduction in parasitaemia. Deltorphin-II in all tested concentrations resisted the progress of infection in a dose-dependent manner, with the maximum percentage reduction of 89.46% at a dose of 0.8 mg/kg/day on day 7 after inoculation (Figure 1). Table 1 shows the average reduction in parasitaemia of all tested doses of deltorphin-II. Infected mice that had been pre-treated with naltriben showed a mean parasitaemia percentage of 4.24%, 7.21%, 10.51%, 39.52% and 63.78% on days 4,7, 10, 14 and 21, respectively (Figure 2).

**Table 1.** Percentage reduction in parasitaemia of *P. berghei*-infected mice treated with deltorphin-II (0.1, 0.2, 0.4 and 0.8 mg/kg/day×4, p.o.), deltorphin-II (0.8 mg/kg/day×4 p.o.) + naltriben (1 mg/kg/day×4, s.c.), chloroquine (8 mg/kg/day) and untreated control group.

Day	Untreated control	Delt 0.1	Delt 0.2	Delt 0.4	Delt 0.8	Delt 0.8 + NTB	CQ 8
4	0	24.44***	47.15***	81.52***	82***	11.29	100***
7	0	40.93***	47.33***	67.37***	89.46***	5.25	100***
10	0	24.93***	48.82***	64.71***	59.17***	11.00	100***
14	0	20.75	25.23	44.42***	51.57***	1.12	100***
21	0	30.15***	27.31*	47.78***	55.01***	-4.39	100***

Values expressed as mean percentage reduction in parasitaemia. Data analysed by one-way ANOVA, ^***^P< 0.001, ^**^ *P*< 0.01, ^*^P< 0.05 compared with control. NTB, naltriben; Delt, deltorphin-II; CQ, chloroquine; p.o., per oral; s.c., subcutaneous.

## 4 Discussion

Malaria chemotherapy has been forced to rely on a limited range of drugs, each with its own pharmacological limitations, of which parasite resistance has been the most damaging. Immunity can also considerably reduce the emergence and spread of resistance. In the present investigation, deltorphin-II exerted a dose-dependent effect on the course of *P. berghei* infection. Administrations of deltorphin-II in *P. berghei*-infected mice caused a significant reduction of parasitaemia levels. Deltorphin-II at all doses tested showed a considerable reduction in parasitaemia, with maximum effects at higher doses (0.4 and 0.8 mg/kg/day). The maximum reduction in parasitaemia of 51% on day 14 was found at a dose of 0.8 mg/kg/day. Subcutaneous administration of naltriben (a potent and selective antagonist for the delta opioid receptor) prior to deltorphin-II administration (0.8 mg/kg/day) erased the protective effect of deltorphin-II. Opioids are known to cause immunomodulation during various microbial and parasitic infections, including malaria [15]. Subtypes of opioid receptors are known to modulate thymic and splenic T-cell proliferation, cytokine production and calcium mobilization [16,17]. The identification and isolation of mRNA encoding the *μ-*opioid receptor gene sequence and expression of kappa (κ) and delta (δ) opioid receptors in human and monkey lymphocytes are evidence for the existence and regulated expression of opioid receptors by cells involved in host defence and immunity [18,19]. However, the available literature shows that the immunomodulatory effects are observed only by δ_2_ receptor agonists but not by δ_1_ [20,21]. Nevertheless, the mechanistic details of the deltorphin-II-induced antimalarial effect should be explored extensively.

## 5 Conclusions

In the present study, naltriben (1 mg/kg/day), a δ_2_-specific antagonist, was found to abrogate the deltorphin-II (0.8 mg/kg/day)-mediated antimalarial effect on elimination of the parasite, thereby suggesting that the effect to be mediated via the δ_2_ receptor. Hence, we suggest that deltorphin-II has a role in suppressing *P. berghei* malaria parasites, possibly by building the immunity through the δ_2_ opioid receptor.
